# ﻿Wing interference patterns are consistent and sexually dimorphic in the four families of crane flies (Diptera, Tipuloidea)

**DOI:** 10.3897/zookeys.1080.69060

**Published:** 2022-01-05

**Authors:** Robert T. Conrow, Jon K. Gelhaus

**Affiliations:** 1 Department of Biodiversity, Earth & Environmental Sciences, Drexel University, 2024 MacAlister Hall Philadelphia, PA, 19104, USA Drexel University Philadelphia United States of America; 2 Department of Entomology, Academy of Natural Sciences of Drexel University, 1900 Benjamin Franklin Parkway, Philadelphia, PA, 19103, USA Department of Entomology, Academy of Natural Sciences of Drexel University Philadelphia United States of America

**Keywords:** Cryptic, Cylindrotomidae, dimorphism, Limoniidae, morphology, Pediciidae, Tipulidae, WIP

## Abstract

Wing interference patterns (WIP) are stable structural colors in insect wings caused by thin-film interference. This study seeks to establish WIP as a stable, sexually dimorphic, species-level character across the four families of Tipuloidea and investigate generic level WIP. Thirteen species of Tipuloidea were selected from museum specimens in the Academy of Natural Sciences of Drexel University collection. One wing from a male and female of each representative species was excised and mounted to a slide with coverslip, placed against a black background, and imaged using an integrated microscope camera. Images were minimally retouched but otherwise unchanged. Descriptions of the WIP for each sex of each species are provided. Twelve of thirteen species imaged had WIP, which were stable and species specific while eight of those twelve had sexually dimorphic WIP. Comparisons of three species of *Nephrotoma* were inconclusive regarding a generic level WIP. *Gnophomyiatristissima* had higher intraspecific variation than other species examined. This study confirms stable, species specific WIP in all four families of crane flies for the first time. More research must be done regarding generic-level stability of WIP in crane flies as well as the role sexual and natural selection play in the evolution of wing interference patterns in insects.

## ﻿Introduction

Wing interference patterns (WIP) were historically known but only recently revealed to be a cryptic physical character of insect wings first reported by Shevtsova et al. (2011). WIP colors and patterns are stable regardless of the angle viewed, so are not iridescent (Shevtsova et al. 2011), but they exist thanks to the same physical property of light that generates iridescence in soap bubbles and oil slicks: thin film interference (Sun et al. 2013). Thin film interference occurs when light enters one of two parallel, thin, non-absorbing (i.e., light) layers, or films, and then bounces between the upper and lower films until exiting through one film (Sun et al. 2013). Thin film interference is most common in the clear wings of insects, with the two thin, chitinous layers forming the parallel thin films (Sun et al. 2013) and 20% of light passing into the wing being reflected between the planes, and exiting through the upper layer (Shevtsova et al. 2011). When the light exits the upper plane of the wing, the thickness of the chitin, as well as the microtopography of the wing at the point of exit will dictate the color observed (Shevtsova 2012). These patterns are created from stable structural properties of the wing, but our ability to observe them may be obscured against white or light-colored backgrounds or enhanced with a black background and bright light perpendicular to the plane of the wing (Shevtsova et al. 2011).

Research suggests that WIP are a novel morphological character that may be sexually selected for in some groups. Cryptic species have been discovered using WIP in two genera of wasps in the family Eulophidae (Shevtsova and Hansson 2011; Hansson and Hambäck 2013). Several works have used WIP as a character in describing a novel species (Buffington 2012; Mitroiu 2013) and as a character in a dichotomous key (Mitroiu 2013; Zhang et al. 2014). Sexual dimorphism of WIP has been documented in many groups of wasps and flies (Shevtsova et al. 2011; Shevtsova and Hansson 2011; Shevtsova 2012) and sexual selection of male WIP by female *Drosophilamelanogaster* Meigen, 1830 has been observed (Katayama et al. 2014; Hawkes et al. 2019).

Most research on WIP in insects has focused on small Hymenoptera and Diptera with clear wings and reduced venation; the result is a continuous pattern across the wing. The existence of WIP in these groups has been well documented in the Cynipoidea (Buffington and Sandler 2011), Eulophidae (Hansson and Shevtsova 2010, 2012) and Drosophilidae (Shevtsova et al. 2011; Katayama et al. 2014; Hawkes et al. 2019). A few studies have looked at larger wasps (Shevtsova et al. 2011; Kangasniemi 2012) or larger flies (Shevtsova et al. 2011; Zhang et al. 2014) or other orders of insects like Odonata (Brydegaard et al. 2018) and Hemiptera (Simon 2013).

Crane flies (Tipuloidea) are one of the largest groups in Diptera with 15,632 recognized species and a global distribution (Oosterbroek 2021). Crane flies vary in wing length from 3 to 36 mm and occupy a diverse set of habitats (Gelhaus and Podenine 2019) and act as a major food source in aquatic and riparian ecosystems (Baxter et al. 2005; Gelhaus and Podeniene 2019). Furthermore, there is evidence of cryptic species within the superfamily (Ujvárosi et al. 2010; Salmela et al. 2014) and a lack of monophyly within the families (Petersen et al. 2010). Tipuloidea is represented in the WIP literature from a single male specimen of Tipula (Savtshenkia) confusa van der Wulp, 1883 presented in Shevtsova et al. (2011). Before that, though, the patterns were mentioned without additional emphasis in at least a few species descriptions of crane flies as early as a century ago (Enderlein 1912). Given the size, diversity, and unresolved phylogeny below the family level, establishment of a novel character such as WIP is essential to our understanding of the group.

Crane flies have relatively large wings with multiple branches of major veins that form upwards of fifteen cells. Additionally, crane flies can have pigment, setae, folds, and reinforcements of the wing surface and veins; all of which play a role in the overall visibility of the WIP (Shevtsova 2012). Crane flies are poorly represented in the current WIP literature; many of the other taxa examined have wings that are small, clear, and with reduced venation. The largest known species of crane fly in North America is *Holorusiahespera* Arnaud & Byers, 1990 (Alexander 1920, as *rubiginosa*) which has a wing length of 40 mm while the smallest species has a wing length of 2.0 mm (de Jong et al. 2007). We suspect given this wide range of wing lengths that WIP will be vastly different among the species of Tipuloidea. Additionally, Shevtsova et al. (2011) showed that in Hymenoptera and Diptera, WIP follow the Newton series: a repeating stable series of color bands known to occur in thin films of between 50 and 1500 nm thickness (Shevtsova et al. 2011). We did not measure wing thickness or map the Newton sequence on our WIP images, but we suspect some of the largest species of the group will have wings too thick to transmit a WIP. If so, we expected to observe opaque, gray wings as described in Shevtsova et al. (2011).

We aim to establish the existence of Wing Interference Patterns across the four families of Tipuloidea using male/female representative pairs and to provide descriptions of the color and pattern of WIP for both sexes for each species. We also seek to establish the stability of WIP within a species and confirm sexual dimorphism in each representative species.

## ﻿Materials and methods

**Figure 1.**
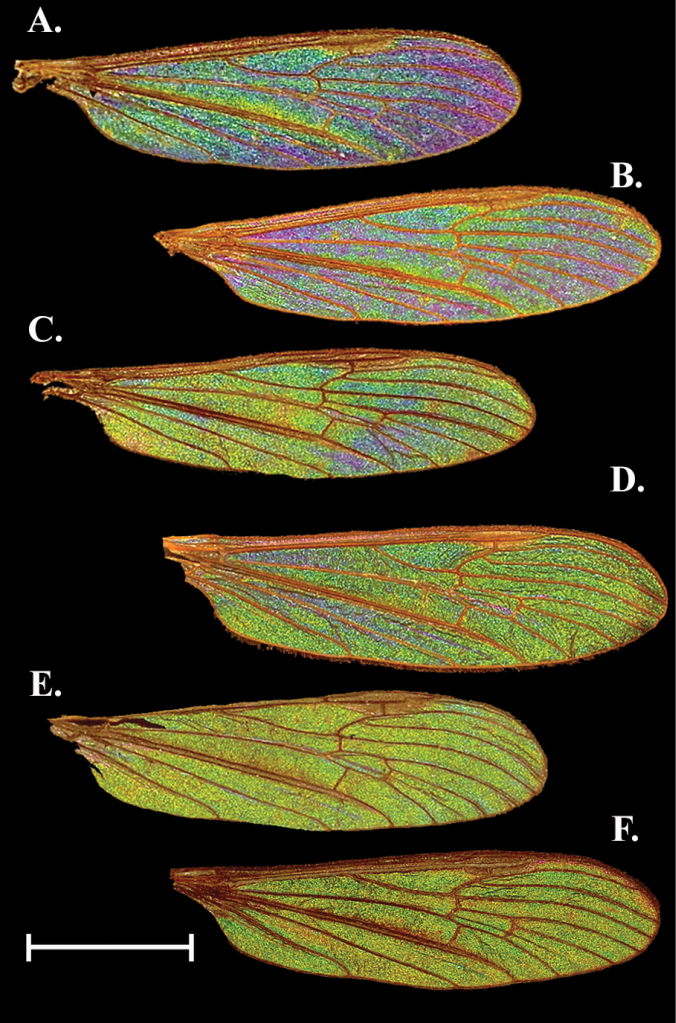
Comparison of the variation in WIP of three female and three male specimens of *Gnophomyiatristissima*. Females examined in this study were found to have a range of WIP from **A** dark blue/ purple **B** blue with mottled yellow **C** green/yellow with hints of blue which appeared most like the male WIP. Males examined also had a range of WIP from **D** green with mottled blue which appeared most like the female WIP**E** solidly green **F** green with mottled magenta. Patterns B and E were the most encountered patterns for females and males, respectively. Scale bars: 1.0 mm.

All specimens were selected from the entomology collection at the Academy of Natural Sciences of Drexel University (**ANSP**) in Philadelphia, Pennsylvania. We examined 45 species (2373 individuals) (Table 1) and selected representative species for each family, with additional species sampled to evaluate generic level WIP relationships (*Nephrotoma spp.*) and to investigate intraspecific variation (*Gnophomyiatristissima* Osten Sacken, 1860) (Table 2). In each representative species both a male and female specimen were selected for comparison. Additionally, both pinned specimens and ethanol-preserved specimens of the same sex and species were compared to determine if preservation had an effect on the stability of WIP. No deviations were seen in WIP color or pattern between same sex specimens of the same species and as such specimens from both preservation methods were used in this study. Preservation type of each specimen used in this study is provided (Table 2). Herein we follow the four-family taxonomy of Oosterbroek (2021). We selected taxa based on availability in the collection and the condition of the wings, as the wings of many pinned specimens were exceedingly brittle and often curled or folded, rendering them unusable for imaging. We specifically chose one species, *Holorusiahespera*, based on size, as we expected this species to have wings too thick to display a WIP. Within each sex for each species, we observed wings across age (1904–2019), geography, and preservation type to confirm stability of the WIP within a species. We found no evidence that any of these metrics had an effect on the stability of WIP. Through this investigation we discovered higher intraspecific variation in *G.tristissima* and chose to image additional specimens for this species (Fig. 1).

**Table 1.** Linked data table of a list of all species of Tipuloidea examined for WIP in this study. The family and valid nomenclature for each species is listed in addition to the presence or absence of a WIP and the number of specimens examined for each species.

Family	Species	WIP present	Number of specimens examined
Cylindrotomidae	*Cylindrotomadistinctissima* (Meigen, 1818)	yes	69
Cylindrotomidae	*Diogmaglabrata* (Meigen, 1818)	yes	7
Cylindrotomidae	*Liogmanodicornis* (Osten Sacken, 1865)	yes	44
Limoniidae	*Eugnophomyialuctuosa* (Osten Sacken, 1860)	yes	3
Limoniidae	*Gnophomyiatristissima* Osten Sacken, 1860	yes	67
Limoniidae	*Gnophomyiacockerelli* Alexander, 1919	yes	29
Limoniidae	*Molophiluspubipennis* Osten Sacken, 1860	yes	32
Limoniidae	*Ormosiaromanovichiana* Alexander, 1953	yes	60
Limoniidae	*Dactylolabiscubitalis* (Osten Sacken, 1869)	yes	18
Limoniidae	*Epiphragmafasciapenne* Say, 1823	yes	123
Limoniidae	*Limnophilamacrocera* (Say, 1823)	yes	78
Limoniidae	*Dicranomyialiberta* Osten Sacken, 1860	yes	113
Limoniidae	*Dicranoptychasobrina* Osten Sacken, 1860	yes	82
Limoniidae	*Elephantomyiawestwoodiwestwoodi* Osten Sacken, 1869	yes	107
Pediciidae	*Pediciaalbivitta* Walker, 1848	yes	26
Pediciidae	*Tricyphonacalcar* (Osten Sacken 1860)	yes	12
Pediciidae	*Tricyphonadegenerata* Alexander, 1917	yes	3
Pediciidae	*Tricyphonaimmaculata* (Meigen, 1804)	yes	18
Pediciidae	*Tricyphonainconstans* (Osten Sacken 1860)	yes	92
Pediciidae	*Ulaelegans* Osten Sacken, 1869	yes	24
Tipulidae	*Phorocteniavittataangustipennis* (Loew, 1872)	yes	3
Tipulidae	*Tanypteradorsalis* (Walker, 1848)	yes	20
Tipulidae	*Dolichopezacarolus* Alexander, 1940	yes	61
Tipulidae	*Dolichopezadorsalis* (Johnson, 1909)	yes	7
Tipulidae	*Dolichopezajohnsonella* (Alexander, 1931)	yes	8
Tipulidae	*Dolichopezaobscura* (Johnson, 1909)	yes	48
Tipulidae	*Dolichopezapolitapolita* (Johnson, 1909)	yes	4
Tipulidae	*Dolichopezatridenticulata* Alexander, 1931	yes	47
Tipulidae	*Brachypremnadispellens* (Walker, 1861)	yes	87
Tipulidae	*Holorusiahespera* Arnaud & Byers, 1990	no	41
Tipulidae	*Nephrotomaferruginea* (Fabricius, 1805)	yes	70
Tipulidae	*Nephrotomamacrocera* (Say, 1823)	yes	106
Tipulidae	*Nephrotomaeucera* (Loew, 1863)	yes	97
Tipulidae	*Nephrotomavirescens* (Loew, 1864)	yes	89
Tipulidae	Tipula (Arctotipula) williamsiana Alexander, 1940	no	55
Tipulidae	Tipula (Beringotipula) borealis Walker, 1848	yes	43
Tipulidae	Tipula (Beringotipula) coloradensis Doane, 1911	yes	75
Tipulidae	Tipula (Lunatipula) atrisumma Doane, 1912	yes	51
Tipulidae	Tipula (Lunatipula) duplex Walker, 1848	yes	101
Tipulidae	Tipula (Lunatipula) valida valida Loew, 1863	yes	47
Tipulidae	Tipula (Pterelachisus) trivittata Say, 1823	yes	106
Tipulidae	Tipula (Trichotipula) oropezoides Johnson, 1909	yes	78
Tipulidae	Tipula (Vestiplex) longiventris Loew, 1863	yes	21
Tipulidae	Tipula (Yamatotipula) sayi Alexander, 1911	yes	52
Tipulidae	Tipula (Yamatotipula) tricolor Fabricius, 1775	yes	49
Total number of specimens examined:			2373

**Table 2.** Linked data table of each specimen image included in this study. The taxonomy and valid nomenclature for each species is listed in addition to the collection location, date, figure reference(s), and preservation type for each specimen.

Specimen code	Species	Sex (M/F)	Location data	Date collected	Figure reference(s)	Preservation type
ANSP-ENT-128038	*Gnophomyiatristissima* Osten Sacken, 1860	F	West Fairmount Park, Philadelphia, PA, USA	1998–06–08	Fig. 1A	Ethanol
ANSP-ENT-128039	*Gnophomyiatristissima* Osten Sacken, 1860	F	West Fairmount Park, Philadelphia, PA, USA	1998–06–08	Figs 1B, 3C	Ethanol
ANSP-ENT-128040	*Gnophomyiatristissima* Osten Sacken, 1860	F	Black Mountains, NC, USA	1912–05–26	Fig. 1C	Pinned
ANSP-ENT-128044	*Gnophomyiatristissima* Osten Sacken, 1860	M	West Fairmount Park, Philadelphia, PA, USA	1998–06–08	Fig. 1D	Ethanol
ANSP-ENT-128043	*Gnophomyiatristissima* Osten Sacken, 1860	M	Tarrytown, NY, USA	1913–06–20	Fig. 1E	Pinned
ANSP-ENT-128041	*Gnophomyiatristissima* Osten Sacken, 1860	M	West Fairmount Park, Philadelphia, PA, USA	1998–06–08	Figs 1F, 3D	Pinned
no code	*Gnophomyiatristissima* Osten Sacken, 1860	F	Montgomery County, MD, USA	2020–06–21	Fig. 9C	none
no code	*Gnophomyiatristissima* Osten Sacken, 1860	M	Montgomery County, MD, USA	2020–06–22	Fig. 9C	none
ANSP-ENT-128045	*Dolichopezaobscura* (Johnson, 1909)	F	South Wales, NY, USA	1911–07–09	Fig. 5C	Pinned
ANSP-ENT-128046	*Dolichopezaobscura* (Johnson, 1909)	M	Black Mountains, NC, USA	1912–06–10	Figs 2, 5D	Pinned
ANSP-ENT-128047	*Cylindrotomadistinctissima* (Meigen, 1818)	F	West Fairmount Park, Philadelphia, PA, USA	1998–07–03	Fig. 3A	Ethanol
ANSP-ENT-128048	*Cylindrotomadistinctissima* (Meigen, 1818)	M	West Fairmount Park, Philadelphia, PA, USA	1998–07–03	Fig. 3B	Ethanol
ANSP-ENT-128049	*Dactylolabiscubitalis* (Osten Sacken, 1869)	F	Black Mountains, NC, USA	1912–05–28	Fig. 4A	Pinned
ANSP-ENT-128050	*Dactylolabiscubitalis* (Osten Sacken, 1869)	M	Black Mountains, NC, USA	1912–05–29	Fig. 4B	Pinned
ANSP-ENT-128051	*Dicranomyialiberta* Osten Sacken, 1860	F	West Fairmount Park, Philadelphia, PA, USA	1998–07–22	Fig. 4C	Ethanol
ANSP-ENT-128052	*Dicranomyialiberta* Osten Sacken, 1860	M	West Fairmount Park, Philadelphia, PA, USA	1998–07–22	Fig. 4D	Ethanol
ANSP-ENT-128053	*Tricyphonainconstans* (Osten Sacken, 1860)	F	West Fairmount Park, Philadelphia, PA, USA	1998–07–22 to 08–16	Fig. 5A	Ethanol
ANSP-ENT-128054	*Tricyphonainconstans* (Osten Sacken, 1860)	M	West Fairmount Park, Philadelphia, PA, USA	1998–07–22 to 08–16	Fig. 5B	Ethanol
ANSP-ENT-128055	*Brachypremnadispellens* (Walker, 1861)	F	West Fairmount Park, Philadelphia, PA, USA	1998–06–20	Fig. 6A	Ethanol
ANSP-ENT-128056	*Brachypremnadispellens* (Walker, 1861)	M	West Fairmount Park, Philadelphia, PA, USA	1998–06–20	Fig. 6B	Ethanol
ANSP-ENT-128057	*Holorusiahespera* Arnaud & Byers, 1990	F	Trout Creek, Juab Co., UT, USA	1922–07–22	Fig. 6C	Pinned
ANSP-ENT-128058	*Holorusiahespera* Arnaud & Byers, 1990	M	Los Padres N.F., San Luis Obispo Co., CA, USA	2019–07–01	Fig. 6D	Pinned
ANSP-ENT-128059	*Nephrotomaferruginea* (Fabricius, 1805)	F	West Fairmount Park, Philadelphia, PA, USA	1998–06–13 to 07–03	Fig. 7A	Pinned
ANSP-ENT-128060	*Nephrotomaferruginea* (Fabricius, 1805)	M	West Fairmount Park, Philadelphia, PA, USA	1998–06–13 to 07–03	Fig. 7B	Pinned
ANSP-ENT-128061	*Nephrotomamacrocera* (Say, 1823)	F	Black Mountains, NC, USA	1912–06–05	Fig. 7C	Ethanol
ANSP-ENT-128062	*Nephrotomamacrocera* (Say, 1823)	M	West Fairmount Park, Philadelphia, PA, USA	1998–06–13 to 07–03	Fig. 7D	Ethanol
ANSP-ENT-128063	*Nephrotomavirescens* (Loew, 1864)	F	West Fairmount Park, Philadelphia, PA, USA	1998–06–13 to 07–03	Fig. 7E	Ethanol
ANSP-ENT-128064	*Nephrotomavirescens* (Loew, 1864)	M	West Fairmount Park, Philadelphia, PA, USA	1998–06–13 to 07–03	Fig. 7F	Ethanol
ANSP-ENT-128065	Tipula (Beringotipula) borealis Walker, 1848	F	Swarthmore, PA	1904–08–27	Fig. 8A	Pinned
ANSP-ENT-128066	Tipula (Beringotipula) borealis Walker, 1848	M	Stony Run Trail, York Co., PA, USA	1998–09–12	Fig. 8B	Pinned
ANSP-ENT-128067	Tipula (Yamatotipula) sayi Alexander, 1911	F	West Fairmount Park, Philadelphia, PA, USA	1998–09–22	Fig. 8C	Ethanol
ANSP-ENT-128068	Tipula (Yamatotipula) sayi Alexander, 1911	M	West Fairmount Park, Philadelphia, PA, USA	1998–09–22	Fig. 8D	Ethanol
no code	Tipula (Yamatotipula) aprilina Alexander, 1918	M	Lindenwold, Camden Co., NJ, USA	2021–04–20	Fig. 9A	none
no code	Tipula (Yamatotipula) aprilina Alexander, 1918	F	Lindenwold, Camden Co., NJ, USA	2021–04–20	Fig. 9B	none
no code	*Ellipteraclausa* Osten Sacken, 1877	unknown	Pioneer, Amador Co., CA, USA	2016–05–27	Fig. 9D	none

Specimens were prepared as in Shevtsova et al. (2011) with certain exceptions. We excised one wing from each specimen, and placed each wing on a slide. Next a drop of 70% ethanol was added to help flatten and orient the wing on the slide and while still wet from the ethanol, a cover slip was carefully placed on top of the wing and slide. We applied gentle pressure to the cover slip to remove air bubbles. Once the ethanol had evaporated the cover slip was adhered to the slide by placing a small drop of Euparal at each corner, making sure that none of the Euparal had seeped onto the wing. Initially we had tested adhesives and fixatives placed over the entire cover slip, but this blocked transmission of WIP.

**Figure 2.**
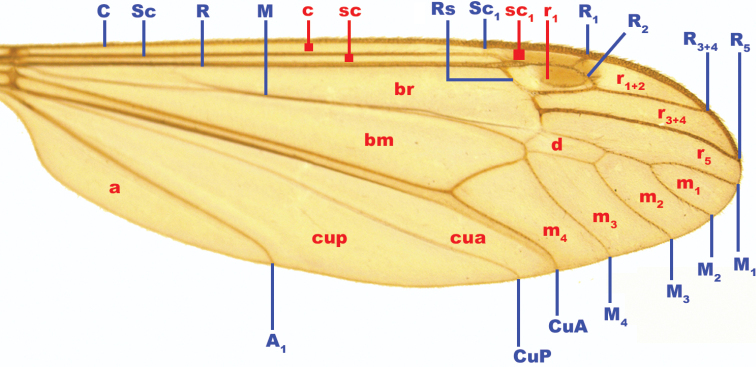
Excised wing of a male specimen of *Dolichopezaobscura* against a white background with notations of wing veins and cells used in this study. Veins are noted in blue with uppercase letters while cells are noted in red with lowercase letters; naming and notations follow those of Saigusa (2006). Abbreviations: **A/a**: anal vein/cell, **bm**: basal medial cell, **br**: basal radial cell, **C/c**: costal vein/cell, **CuA/cua**: anterior cubitus vein/cell, **CuP/cup**: posterior cubitus vein/cell, **d**: discal cell, **M/m**: Medial vein/cell, **R/r**: radial vein/cell, **Rs**: radial sector vein, **Sc/sc**: subcostal vein/cell. Image not to scale.

A black background was created using light-absorbing black-out fabric with adhesive backing from Edmund Optics (Item #54–585). This fabric was placed beneath each wing slide prior to imaging. Imaging for all but three species was performed using a Leica S9i stereomicroscope (Model AF6000). Images for three species (Tipula (Yamatotipula) sayi Alexander, 1911, Dolichopeza (Oropeza) obscura (Johnson, 1909), and *Nephrotomamacrocera* (Say, 1823)) were too distorted to be used and were reshot using a Leica (Model EZ4 D) stereomicroscope with an integrated 3mp camera. Each specimen was imaged using LEICA APPLICATION SUITE X (version 3.0.11.20652). Files were saved in .tiff format and edited in ADOBE PHOTOSHOP (version CS6). Alterations in Photoshop were restricted to increasing the saturation by no more than 10%, reducing brightness by up to 20%, increasing the contrast by up to 20%, cropping the wing from the background, darkening the background, and using the spot-healing tool to remove dust and debris as needed. We follow the four-pattern concept of WIP put forth in Buffington and Sandler (2011): campiform (WIP of mostly one color, usually blue), galactiform (mottled patches and swirls of color like the spirals of galaxies), radiform (radial bands emerging from the medial sector and expanding in concentric bands to the margin), and striatiform (longitudinal bands of color that often follow anal veins). Wing definitions follow those of Saigusa (2006) (Fig. 2).

Descriptions of WIP herein are an attempt to provide a written account of the WIP across the entire wing. This was difficult to capture in a single image, mostly due to the size of many wings as well as the folds and textured surfaces of the wings of many crane flies examined. In the larger species, the veins themselves were thick enough to keep the wing from lying flat on the slide. Rather than making composite images we attempted to provide as complete a WIP as possible in a single image. As such, cells adjacent to the costal margin (especially c, sc_1_, and sc_2_ that in many taxa are exceedingly narrow) often have WIP that are distorted by corrugation, folds, and pigment. We have still attempted to provide those details in the descriptions and have noted in parentheses behind the given characters when they are not visible in the corresponding figure though WIP should be visible when observed in person.

### ﻿Abbreviations

**A/a** anal vein/cell;

**ANSP**Academy of Natural Sciences of Drexel University;

**bm** basal medial cell;

**br** basal radial cell;

**C/c** costal vein/cell;

**CuA/cua** anterior cubitus vein/cell;

**CuP/cup** posterior cubitus vein/cell;

**d** discal cell;

**M/m** Medial vein/cell;

**R/r** radial vein/cell;

**Rs** radial sector vein;

**Sc/sc** subcostal vein/cell;

**WIP** Wing Interference pattern(s).

## ﻿Results

### ﻿WIP descriptions

**Family Cylindrotomidae**

#### ﻿ Cylindrotoma distinctissima

Taxon classificationAnimaliaDipteraCylindrotomidae

(Meigen, 1818)

3EBAC4A8-8FC6-54DA-B58C-82D07D802684

Fig. 3

##### General appearance.

In both sexes, the basal half of the wing green to green-yellow (with males being greener and females brighter and more yellow) with swirling magenta striations and beyond the cord a mostly magenta apical half forming a patch or spot. Males with magenta bands reduced before the cord and expanded beyond. Generally, males appear more magenta at a distance while the females appear more banded red/green. The pattern is striatiform before the cord and galactiform after.

**Figure 3.**
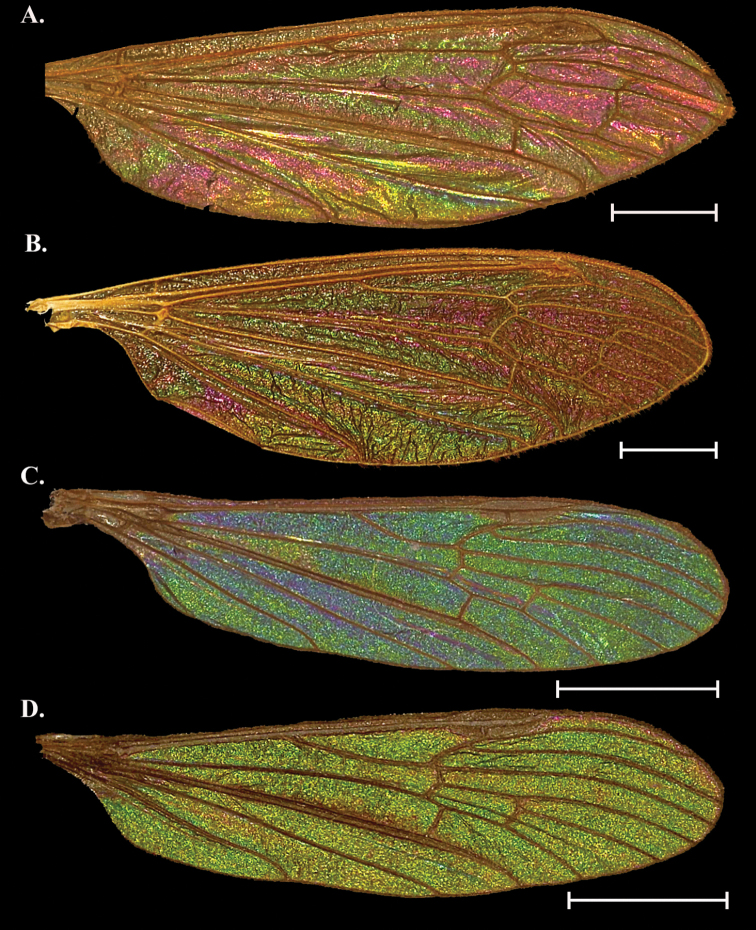
Wing Interference Pattern on excised wings of male/ female pair of two species of Tipuloidea. Excised wings of a male/ female pair of two species of crane flies. Wings were excised, flattened between a glass slide and cover slip, and photographed under a microscope using transmitted light **A***Cylindrotomadistinctissima* female **B***Cylindrotomadistinctissima* male **C***Gnophomyiatristissima* female **D***Gnophomyiatristissima* male. Scale bars: 1.0 mm.

##### Female description

**(Fig. 3A).** Cells c, sc_1_, and sc_2_ with WIP obscured by wing topography but can appear as a mottled green/ magenta (not visible in Fig. 3); cell r_1_ green with a magenta band through the center, and distal edge of cell obscured by pterostigma pigment, which may bleed into nearby cells. All other cells r and m, as well as d, show strong magenta in the center with various-sized patches of green edging the margins. Cells cua, cup, bm, and br with large striations of green and magenta with many stretching over more than one cell; the green is generally kept to the center of the cell. The anterior edge of cup has a small strip of indigo edging vein CuA and green coloration in cells a_1_, cup, and cua appearing to bleed into yellow. Apical edge of cell A_1_ with magenta where vein A meets the wing margin, in center of cell three magenta spots surrounded by green, magenta may also edge the margin.

##### Male description

**(Fig. 3B).** Similar to female in color and pattern but magenta bands reduced before the cord and expanded after.

##### Notes.

There can be some subtle pattern differences in males of this species but they do not appear to be sexually dimorphic as this variation is inconsistent.

###### Family Limoniidae

#### ﻿ Gnophomyia tristissima

Taxon classificationAnimaliaDipteraCylindrotomidae

Osten Sacken, 1860

F26BF33A-E2E8-5E90-8D44-1B52BA703CD0

Figs 1 , 3

##### General appearance.

Sexually dimorphic. Female with galactiform dark blue patches over a green background with some yellow-green patches. Male a bright yellow green galactiform, occasionally with faint magenta patches/bands. This species had higher intraspecific variation and variation within the sexes than any other species examined.

##### Female description

**(Figs 1A–C, 3C).** Cell c and sc with WIP obscured by wing topography but often with mottled magenta overall (1A, D, not visible in Fig. 3). A thin band of yellow green can be seen lining the pterostigma pigmentation posteriorly; the band usually originates at the proximal edge of the pterostigma, staying rather regular in width following R through the r_1_, widening as it reaches R_2_ and passing into cells r_3_ and r_2_, then looping around and ending where R_1_ meets the margin. This color is highly variable and can exist as nothing more than a hint of yellow or expanded wider; it may follow the wing margin at least through r_2_ if not further to the posterior wing margin. All r and m cells, as well as cell d uniform blue green, with purple lining the margins (the width of these purple bands is highly variable). Cells cua, cup, a_1_, bm, and br with a similar pattern to the apical cells but with less purple overall. Cell cua with a large fold that is usually purple (occasionally yellow). A second fold originates in br, follows into d and ends in cell m_2+3_.

##### Male description

**(Figs 1D–F, 3D).** Male WIP generally yellow green campiform with some degree of blue and yellow but patterns as in female, band originating at pterostigma magenta, folds in basal cells blue.

##### Notes.

Variations in the WIP of *G.tristissima* were greater than the intraspecific variation seen in other species examined in this study. Females and males both appeared to have a gradient. The darkest females (Fig. 1A) appear almost indigo near the margin and basal cells a mottled green blue while the lightest wings (Fig. 1C) have blue reduced throughout and only prominent near the cord and generally appearing more like a male wing. The most common female WIP configuration (Fig. 1B) is a solid blue to blue green with only hints of yellow around the cord. Males have a similar gradient and the darkest male WIP have flares of blue as in light females (Fig. 1C). The most common males are solidly green (Fig. 1E) but may have some hints of magenta tracing veins. A third type of male WIP are similar to the common pattern but with magenta regions expanded (Fig. 1F). Occasionally small striations like those around the pterostigma can be inverted so females have a magenta band and males with a yellow-green band.

#### ﻿ Dactylolabis cubitalis

Taxon classificationAnimaliaDipteraCylindrotomidae

(Osten Sacken, 1869)

AB285A1B-120E-519E-803B-985E166BB461

Fig. 4

##### General appearance.

Sexually dimorphic. Wing galactiform with large green splotches and thin magenta striations originating from the base of the wing moving outward. Both sexes have a magenta band near the center of the wing but the magenta occupies different cells in males and females.

**Figure 4.**
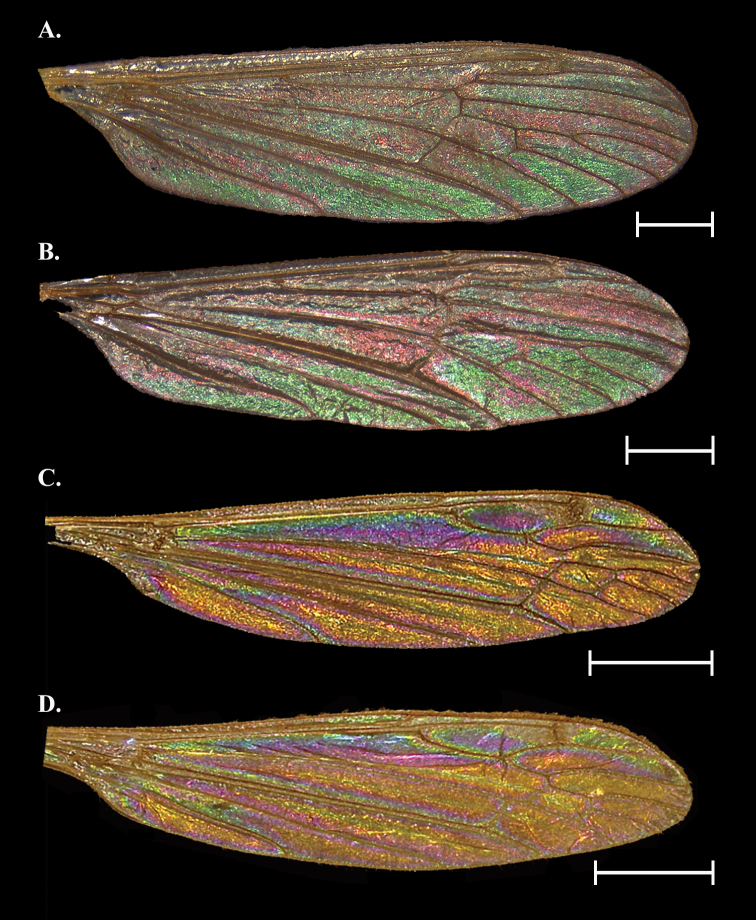
Wing Interference Pattern on excised wings of male/ female pair of two species of Tipuloidea**A***Dactylolabiscubitalis* female **B***Dactylolabiscubitalis* male **C***Dicranomyialiberta* female **D***Dicranomyialiberta* male. Scale bars: 1.0 mm.

##### Female description

**(Fig. 4A).** Cell r_1_ and r_1+2_ both with a band of magenta following the posterior edge with the rest of the cell a mottled magenta and green where visible. Cell r_3_ mostly green, cell r_4_ mostly green with an oblong spot of magenta in the center of the cell. Cells r_5_, m_1_ to m_4_, and d forming a multi-cell pattern featuring a green splotch centered on m_3_ and reaching into m_1_ and m_5_ to either side. Each cell also has magenta lining the margin. Cells cua, cup, and a_1_ with magenta striations originating from the wing origin and ending at the anal margin and generally paralleling veins. Cell bm with a green center, br like bm but with green center faint.

##### Male description

**(Fig. 4B).** Pattern similar overall to female with these exceptions: cells r_3_ and r_4_ with magenta expanded while r_5_ has greener than in female. It is as though the magenta patterning in the female has been shifted anteriorly by one cell in the radial cells. The multi-cell pattern across median cells is inverse of female with a magenta spot situated in cells m_3_ and m_4_ centered on M_4_. All basal cells as in female.

##### Notes.

Both sexes have a “spot” near the apex of the wing that originates on either edge of r_4_ about halfway between the basal edge and the wing margin. In the female this is a barely visible magenta spot, but in the male this spot expands to fill most of r_4_ as well as part of r_3_ and r_5_. All cells basal to the cord are similar between the sexes. Pattern galactiform with striatiform portions present.

#### ﻿ Dicranomyia liberta

Taxon classificationAnimaliaDipteraCylindrotomidae

Osten Sacken, 1860

293D42E9-E48E-5835-85B8-8513C32E8C2C

Fig. 4

##### General appearance.

Pattern is a bold galactiform containing almost the entire spectrum of colors found in WIP. Anterior cells with large bold blue to purple centers encircled by green, yellow, and magenta in that order.

##### Female description

**(Fig. 4C).** Cell c, sc, and sc_1_ obscured by wing topography but can appear as mottled green/ magenta (not visible in Fig. 4C). Pterostigma obscures distal half of cell r_1_ and the proximal edge of cell r_1+2_. Cell r_1_ otherwise with magenta splotch at center surrounded by a narrow blue band (the combination of which appears purple) then a green oval surrounding that becoming yellow at the margin of the cell. Cell r_1+2_ with pattern similar to r1 but with hints of magenta at the margin. Cell r_3+4_ with bright yellow oval at proximal end, encircled by a magenta band, followed by blue, green, yellow, and magenta bands. Cells r_5_, m_1_ to m_3_, and d campiform yellow with small green striations through the center of cells or magenta directly adjacent to the veins. Cells cua, cup, a_1_, and bm also campiform yellow but with bright striations of green, magenta, and blue tracing the veins and following to the wing margin. Cell br with a large magenta spot in the center of the cell and tapering to the proximal and distal ends. On the proximal half the magenta is surrounded by blue, green, and yellow bands while on the distal edge the blue band gives way to a magenta band followed by a golden yellow band.

##### Male description

**(Fig. 4D).** Almost identical to female, but may be a bit dull as compared to female WIP.

##### Notes.

The WIP of this species is one of the most colorful we encountered in this study, and one of the few containing yellow as a dominant color. The WIP in *D.liberta* does not appear to be sexually dimorphic and differences between the sexes are not consistent.

###### Family Pediciidae

#### ﻿ Tricyphona inconstans inconstans

Taxon classificationAnimaliaDipteraCylindrotomidae

(Osten Sacken 1860)

4E6915E5-E416-597E-B41B-764FB8704CB3

Fig. 5

##### General appearance.

Sexually dimorphic. Pattern striatiform in basal cells and galactiform in apical cells. Both sexes have basal half of the wing green with magenta striations including one that extends to the margin. Two spots occur on the posterior half of the wing. In females these spots are yellow to yellow-green but in males the spots are blue to purple-magenta.

**Figure 5.**
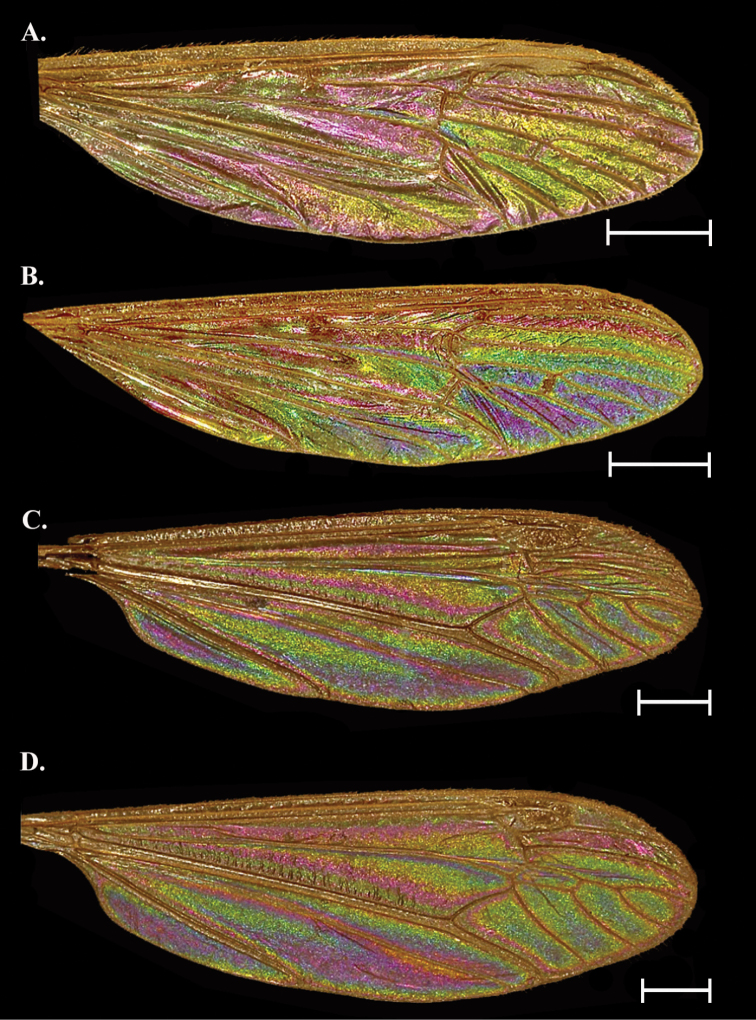
Wing Interference Pattern on excised wings of male/ female pair of two species of Tipuloidea**A***Tricyphonainconstansinconstans* female **B***Tricyphonainconstansinconstans* male **C***Dolichopezaobscura* female **D***Dolichopezaobscura* male. Scale bars: 1.0 mm.

##### Female description

**(Fig. 5A).** Cells c, sc, sc_1_, r_1_, and r_1+2_ obscured by topography/ pigmentation though r_1_ and r_1+2_ with mottled magenta and green, though this may or may not be visible. Cells r_3_ and r_4_ mostly magenta with a ribbon of light green on apical edge of r_3_. A magenta band starts in r_3_ and following the posterior wing margin to at least cell cua. Cells r_5_ to m_4_ and d forming a large, light green circle with the outer margins of the outer cells continuing the magenta strip at the margins. Cell cua and cup with a light green oval centered over CuP and surrounded by magenta. Cells bm and br mostly magenta with the basal sections green.

##### Male description

**(Fig. 5B).** Similar to female with the following exceptions: Cell r_4_ green/yellow, all m and d cells a deep magenta/purple at center, followed by a thin ring of blue, green, and yellow at the margins. The band following the posterior margin yellow, spot centered on CuA with magenta center encircled by concentric blue, green, yellow, and magenta bands. Cell a_1_ as in female but magenta reduced. Cell bm as in female but color inverted.

##### Notes.

Color but not pattern is dimorphic, and males are distinct in having a bright blue spot centered in the m cells while females lack almost any blue coloration, instead having the spot green. The same is the case with the spots on cua and cup. In males, these blue patches are in stark relief to the magenta/green of the wing.

###### Family Tipulidae

#### ﻿ Dolichopeza obscura

Taxon classificationAnimaliaDipteraCylindrotomidae

(Johnson, 1909)

416F1963-60FA-5023-9CAE-8BC63CF177B6

Fig. 5

##### General appearance.

Sexually dimorphic. Bright and colorful galactiform patterns with striatiform near the base. Three large magenta striations starting near wing origin, two of which terminate at the cord, the anterior-most band continues to the margin. Two large blue to purple-magenta spots near posterior margin, otherwise wing cells green with faint blue centers and sometimes magenta lining the margins.

##### Female description

**(Fig. 5C).** Cells c and sc with deep ridges/baffles but with spots of magenta and green (not visible in Fig. 5C). Cells r_3_ and r_4+5_ mostly green with a magenta striation through the middle of each cell. All m cells as well as cell d with large blue/purple spots in the center that transition to green, followed by concentric rings of yellow and magenta to the margins that gives the effect of a rusty brown WIP. Cells cua and cup with a magenta spot that forms on either side of vein CuP, close to where the vein reaches the margin. The magenta bleeds into both cells, transitioning to blue/purple, then green, and finally yellow near the basal edge of the cell. This “spot” occupies most of the distal portion of the cells. Cell a_1_ with a large magenta spot in the center, surrounded by concentric rings of blue/purple, green, yellow, and magenta touching the margins. Cells bm and br with three striations running parallel to M and CuA, respectively. The striations from posterior to anterior are green, magenta, and yellow. A blue streak parallels M in bm,

##### Male description

**(Fig. 5D).** As in the female with the following exceptions: Cells r_5_ and m_1_ to m_4_ with magenta and blue/ purple centers reduced to small spots. Magenta spot between cua and cup expanded, filling more than half the distal halves of the cells. The magenta spot in cell a_1_ is similarly expanded, with only a small band of green/yellow at the margins. The blue streak in br is expanded to a large blue/purple striation situated apically from the yellow band.

##### Notes.

Much like *C.distinctissima*, it is unclear if the differences in *D.obscura* are due to plasticity or true sexual dimorphism. The pattern and most colors are identical, though the differences in the male (noted above) do appear more substantial, we still consider this species to lack sexually dimorphic WIP.

#### ﻿ Brachypremna dispellens

Taxon classificationAnimaliaDipteraCylindrotomidae

(Walker, 1861)

B88E3AB7-0377-5DF4-B4E5-73FF18F6D169

Fig. 6

##### General appearance.

Sexually dimorphic. Females with green and magenta striations before the cord and cells with green centers and magenta edges beyond the cord. Males have nearly identical patterns, but all instances of magenta and green are inverted. Cells beyond the cord galactiform.

**Figure 6.**
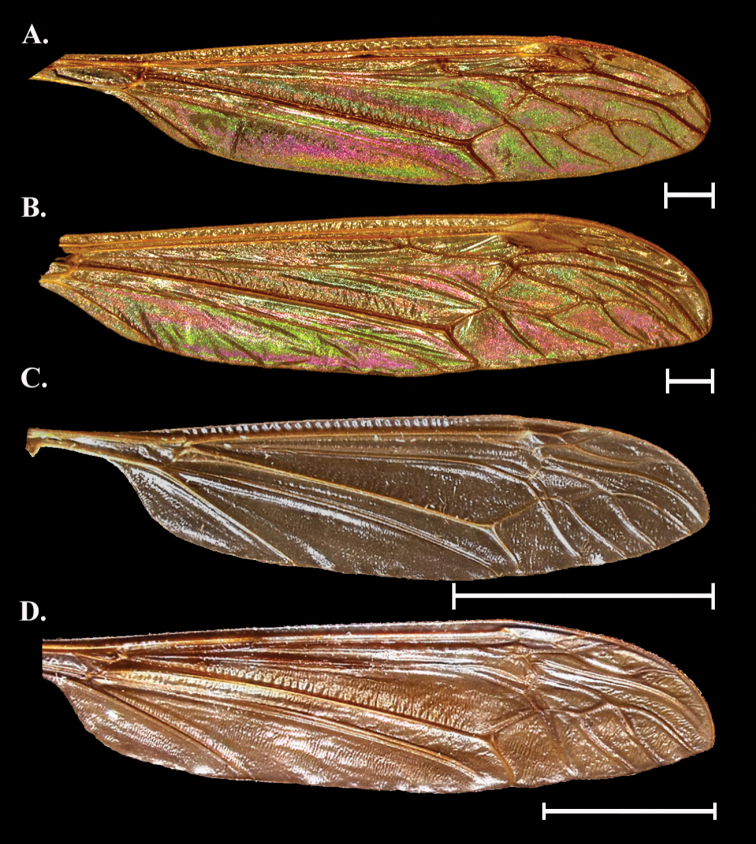
Wing Interference Pattern on excised wings of male/ female pair of two species of Tipuloidea**A***Brachypremnadispellens* female **B***Brachypremnadispellens* male **C***Holorusiahespera* female **D***Holorusiahespera* male. Scale bars: 1.0 mm (**A, B**), 1.0 cm (**C, D**).

##### Female description

**(Fig. 6A).** Cells c and sc obscured by ridging/thickness of wing, though small patches of magenta or green may show through (not visible in Fig. 6A). Cells sc_1_, sc_2_, and distal half of r_1_ with WIP obscured by pterostigma; proximal half of r_1_ with green center ridged by magenta. Cell r_1+2_ with magenta center surrounded by green. Cell r_3_ with a similar pattern as r_1+2_, though pattern is more striated in r_3_. Cells r_4+5_, m_1_ to m_4_, cua, br, and d with center green and magenta lining the margin of each cell, though magenta can be broken or uneven and invade the green region. Cells a_1_, cup, cua, and bm with magenta center and green edging the margin, can also be broken or invading the magenta region.

##### Male description

**(Fig. 6B).** As in the female wing with the following differences: All r cells with green center and magenta edging the margin; patterns as in female. All m cells, cua, and a_1_ with center magenta and green invading the magenta region; patterns as in female. Cells cup, bm, and br with green centers and magenta edging margins; patterns as in female.

##### Notes.

This is the clearest example in the taxa studied where the WIP patterns are identical between males and females but with the magenta and green regions inverted almost exactly.

#### ﻿ Holorusia hespera

Taxon classificationAnimaliaDipteraCylindrotomidae

Arnaud & Byers, 1990

44B49904-C99E-52C2-82E5-233FD8D4543E

Fig. 6

##### General appearance.

WIP absent. Appearance is a glossy opaque amber color.

##### Female description

**(Fig. 6C).** Wing lacks any WIP. All cells are a uniform tan-amber in pigment, but no interference pattern is transmitted. Wing surface is heavily textured and ridging and folds are visible across the surface of the wing.

##### Male description

**(Fig. 6D)**. Wing is similar to female and lacks any WIP.

##### Notes.

As noted in Shevtsova et al. (2011) WIPs become obscured as wing thickness approaches 1500 nm. While we did not measure the thickness of wings for this study, the size of *H.hespera* suggests the thickness of the wing is a likely reason for the absence of WIP.

#### ﻿ Nephrotoma ferruginea

Taxon classificationAnimaliaDipteraCylindrotomidae

(Fabricius, 1805)

394EC9D2-6327-5E94-AC9A-52FFFC95F07E

Fig. 7

##### General appearance.

Sexually dimorphic. Pattern is striatiform but cells beyond the cord campiform green with magenta spots and striations. Both sexes with four large magenta striations starting near the origin and terminating at the cord; the anterior-most striations continue past the cord to near the margin; magenta striations are larger and brighter in males. Wings have a glossy sheen to them that causes the WIP to seem slightly washed out or glass-like.

**Figure 7.**
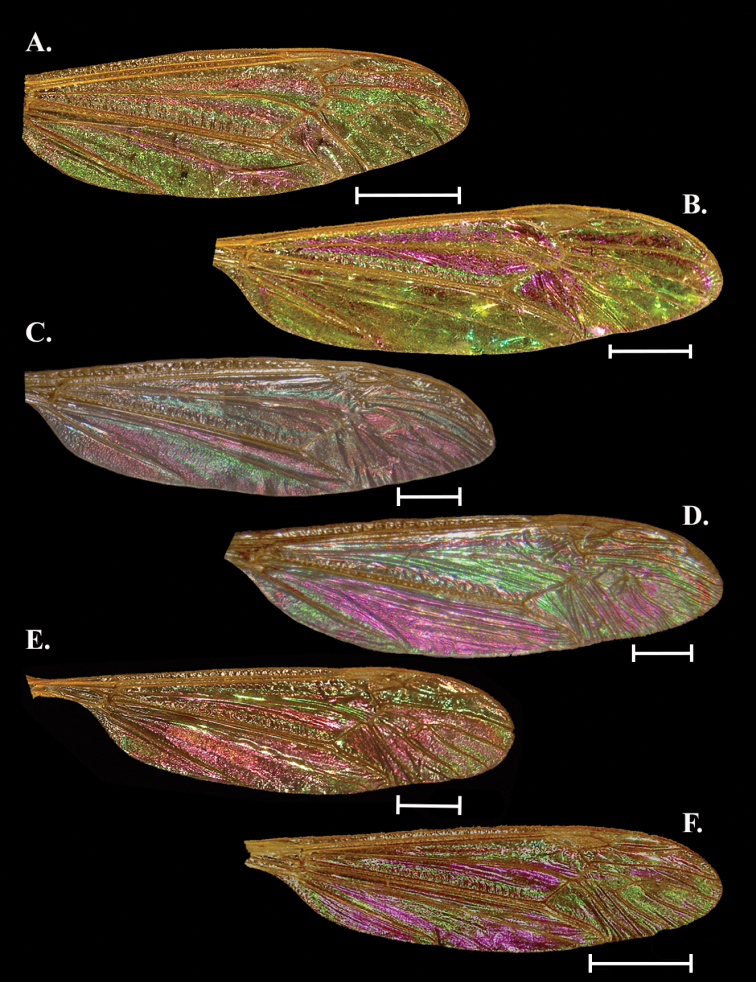
Wing Interference Pattern on excised wings of male/ female pair of two species of Tipuloidea**A***Nephrotomaferruguina* female **B***Nephrotomaferruguina* male **C***Nephrotomamacrocera* female **D***Nephrotomamacrocera* male **E***Nephrotomavirscens* female **F***Nephrotomavirscens* male. Scale bars: 1.0 mm.

##### Female description

**(Fig. 7A).** Cells c, sc, r_1_, and r_1+2_ obscured by topography and pigment. Cell r_3_ green-magenta band near the center. Cell r_4_ with most of the center magenta and green to the margins; a small striation breaks vein R_5_ and enters r_5_, ending at the wing margin near the end of vein M_1_. Cells r_5_, m_1_ to m_3_, and d solidly green, with small flares of magenta at the margins; cell m_4_ with a magenta spot in the center and green to the margins. All basal cells green with magenta striations following veins.

##### Male description

**(Fig. 7B).** As in female but with magenta expanded anteriorly and reduced posteriorly.

##### Notes.

*Nephrotomaferruginea* appeared to have less intraspecific variation in WIP than others based on the large number of specimens examined. Patterns are similar but colors sexually dimorphic. The WIP in this species is difficult to capture as a full pattern due to natural folds in the wing and males especially can look glassy or washed-out.

#### ﻿ Nephrotoma macrocera

Taxon classificationAnimaliaDipteraCylindrotomidae

(Say, 1823)

41EA4912-5213-5AD3-88B1-02E832C3EB4F

Fig. 7

##### General appearance.

Sexually dimorphic. Female wing mottled green and magenta striatiform before the cord while magenta predominates beyond. Male wing mostly green overall with a clear magenta spot centered around m cells and most of cua and cup magenta. Striatiform pattern with galactiform portions beyond the cord.

##### Female description

**(Fig. 7C).** Cells c, sc, r_1_, and r_1+2_ obscured by topography and pigment. Proximal r cells mainly green with small magenta striations. Cells m_1_ to m_3_ and d solidly magenta, with small flares of green at the margins; cell m_4_ green with an indistinct magenta striation stretching diagonally from the end of M_4_ to the origin of the M-Cu cross vein. Basal cells with almost alternating magenta and green striations.

##### Male description

**(Fig. 7D).** Cells r_5_, m_1_ to m_4_, and d green but with a large generally oval magenta spot centered around m_2_ and m_3_. Cells cua and cup a bold magenta with only a faint trace of green at the exterior margins of each cell. Cell a_1_, bm, and br much greener than female.

##### Notes.

Males and females are similar in that the color is predominantly green and magenta but there is some inversion of a pattern. Some cells look similar, others with color inverted, and some cells completely different between the sexes. The result is a very showy male wing with big blocks of color while the female has a more subdued, mottled look.

#### ﻿ Nephrotoma virescens

Taxon classificationAnimaliaDipteraCylindrotomidae

(Loew, 1864)

8BE51ADC-8B0A-5846-A980-4416A4FEA248

Fig. 7

##### General appearance.

Sexually dimorphic. Prior to the cord, wings with wide magenta bands in both sexes. Females are mostly magenta beyond the cord with green in the anterior most cells. Males mostly green beyond cord with a magenta spot near the anterior distal margin. Patterns are galactiform beyond the cord and striatiform before it, though striations are seen after the cord as well.

##### Female description

**(Fig. 7E).** Cells c, sc, r_1_, and r_1+2_obscured by topography and pigment. Cells r_3_ and r_4_ mostly green, cells r_5_, m_1_ to m_4_, and d magenta, but with green striations and a green spot centered in m_3_. Basal cells with large magenta striations filling most of the cells and small green striations between them. A green spot sits on CuP and crosses into cua and cup.

##### Male description

**(Fig. 7F).** Similar to female pattern but some colors different or inverted. Proximal r cells, all m cells, and cell d opposite to female, with more magenta anteriorly and mostly green posteriorly. Most cells prior to the cord as in female but some colors inverted. Also, green spot centered on CuP expanded and magenta in males.

##### Notes.

As in *Nephrotomamacrocera*, this species shows male and female wings with similar WIP patterns but inverted colors. Male wings are distinctly green with small striations and spots of magenta while female wings have a magenta base with green striations and spots.

#### ﻿ Tipula (Beringotipula) borealis

Taxon classificationAnimaliaDipteraCylindrotomidae

Walker, 1848

BA662B98-D17B-59C3-B090-EF98D31FF095

Fig. 8

##### General appearance.

WIP similar in both sexes but with variously sized and spaced pigment clouds of grey and brown. Otherwise WIP dull, mostly green with clouds of magenta. Pattern mostly galactiform with some striations on the basal half of the wing.

**Figure 8.**
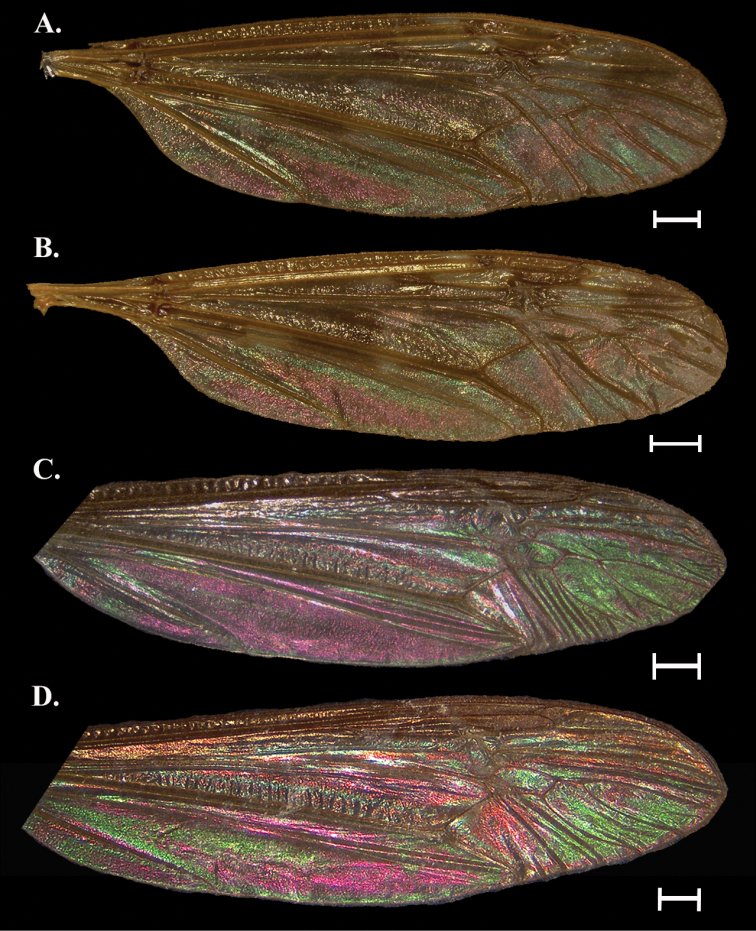
Wing Interference Pattern on excised wings of male/ female pair of two species of Tipuloidea**A**Tipula (Beringotipula) borealis female **B**Tipula (Beringotipula) borealis male **C**Tipula (Yamatotipula) sayi female **D**Tipula (Yamatotipula) sayi male. Scale bars: 1.0 mm.

##### Female description

**(Fig. 8A).** Cells c, sc, r_1_, and r_1+2_ obscured by pigment and texture. A large brown cloud of pigment centered in the middle of r_3_ obscuring WIP in cells r_2_, r_3,_ and cell r_4+5_ but generally both cells green near the base and magenta near the margin. Cell r_4+5_ green with magenta lining the margins and a magenta band through the center of the cell. All m cells with a magenta band that traces the margin and a larger band near the apical third of the cell that cuts across each cell and joins the center magenta band in r_4+5_; cell d green with magenta center. Cell cua partially obscured with pigment but generally green with magenta at margins, cup and a_1_ green with large magenta centers and magenta lining the margins. Both bm and br with pigment partially blocking WIP but appear to follow patterns of other cells with large green patches and smaller magenta bands near margins.

##### Male description

**(Fig. 8B).**WIP as in female, with some magenta regions more pronounced than the female pattern.

##### Notes.

WIP patterns are stable in this species, but the placement of the wing pigment is variable. This means the pigment can obscure portions of the WIP which may superficially appear like the WIP is unstable. Like other species in this study, *T.borealis* males have the same pattern as females but with expanded magenta regions. This does not seem to be dimorphic, but more study is needed.

#### ﻿ Tipula (Yamatotipula) sayi

Taxon classificationAnimaliaDipteraCylindrotomidae

Alexander, 1911

2BAA8FCA-5CF6-5F9B-8E25-5916FF67AA06

Fig. 8

##### General appearance.

Sexually dimorphic. Patterns similar but with male magenta regions expanded or reduced compared to females. Overall wings appear green with magenta striations, but lower halves of wing tip in both sexes solidly green.

##### Female description

**(Fig. 8C).** Cells c, sc, r_1_, r_1+2_, and the anterior half of r_2_ obscured by a dark pigmentation band that runs parallel to the apical wing margin. The basal portion of r_1_ with magenta anteriorly and green posteriorly. All proximal r cells, m_1-3­_, and cell d forming a solid green field with two magenta striations anteriorly and small red spots posteriorly. Basal cells all mostly magenta with narrow green striations.

##### Male description

**(Fig. 8D).**WIP as in female with following exceptions: all r cells with more magenta than female, all m cells with more magenta than female, cua and cup greener than in female, br and bm with more magenta than female.

##### Notes.

This species has a subtle dimorphism. Like others, the pattern between the sexes is similar, but with pattern deviations and color inversions. Wings have deep folds and as such WIP can be obscured, especially in dried specimens.

## ﻿Discussion

We confirm stable, structural Wing Interference Patterns (WIP) in the four families of Tipuloidea. Despite small deviations the overall patterns were stable within each sex and/ or species and were not affected by age of specimen, location collected, or method of preservation. Twelve of the thirteen species sampled had a distinct WIP across the entire wing surface. The sole exception was *Holorusiahespera*, which lacked a WIP, likely due to the size and thickness of the wing as we predicted (Fig. 6C, D). This agrees with the findings of Shevtsova et al. (2011) who noted that the WIP of flies follow the Newton sequence, and wings thicker than 1500 nm will lack a WIP and appear opaque gray. We found eight of the twelve species with a WIP were sexually dimorphic in color if not pattern; *Cylindrotomadistinctissima* (Fig. 3A, B), *Dicranomyialiberta* (Fig. 4C, D), *Dolichopezaobscura* (Fig. 5C, D), and *Tipulaborealis* (Fig. 8A, B) lacked sexually dimorphic WIP.

We have demonstrated that WIP are stable within each sex and each species. In ten of twelve species there were minimal variations in WIP consistent with phenotypic variation. Because WIP color is determined by the nanometer-level thickness of the chiton layer, one could expect that among individuals of the same sex there would be some degree of variation in wing thickness. Indeed, these results support the findings of Shevtsova et al. (2011) and Shevtsova and Hansson (2011) who both noted patterns are more stable than color or hue of WIP. We also provide the first cell-by cell descriptions of WIP as a diagnostic character. One species, *Gnophomyiatristissima*, showed increased variation in color and pattern relative to the other species we examined. Both males and females showed this variation and there appeared to be a gradient of WIP color. Additionally, some males and females showed an almost inverted WIP. More work is needed to understand if this is simply a gradient of wing thickness or if there is a selection force acting on the WIP in this species.

Sexual dimorphism of WIP in crane flies is clear and common in the species we examined. While there is no documented evidence of sexual selection of WIP in Tipuloidea, female choice of WIP has been demonstrated in various species of *Drosophila* (Ala-Honkola and Manier 2016; Hawkes et al. 2019) including evidence that females use visual cues in choosing mates and prefer winged males (Watanabe et al. 2018). Further, Katayama et al. (2014) found that females of *D.melanogaster* preferred wings of mates with a high degree of saturation and a centrally stable hue. Even with our limited sampling of species, many of the sexually dimorphic species in our study had males with bolder, more contrasted colors than females. *G.tristissima* is a widespread species (Alexander 1920) and as such we may be seeing the results of female choice of WIP or a geographic effect. A larger study of this species should be done to try and gauge the level of variation as well as the existence of sexual selection for WIP in *G.tristissima*. Butterworth et al. (2021) performed quantitative, viewer independent methods to evaluate WIP in Calliphoridae, a method that is well suited for examining the variation in *G.tristissima* as well as further WIP studies in Tipuloidea.

Our work suggests that WIP are a stable, reliable species level trait in Tipuloidea and this agrees with the recent WIP literature (Shevtsova et al. 2011; Shevtsova and Hansson 2011; Zhang et al. 2014; and Butterworth et al. 2021). In Tipuloidea, traditional identification of species is often based on male genitalic features, with females in some groups being difficult to identify to species, and even identification keys to the species level are not available for the female stage for many genera. The WIP may provide a useful set of features for separating very similar species in the female stage, e.g., in Tipula (Beringotipula) separation of the over 20 species in North America at present is based on male genitalic features only.

Additionally, we did not see evidence of a generic level pattern among the three species of *Nephrotoma* examined, although all three species generally had green/magenta striated wings. We are aware of two studies to examine generic level WIP (Buffington and Sandler 2011; Shevtsova and Hansson 2011) both of which found some suggestion of a generic level WIP in certain taxa. We sampled only three of 484 species in the genus *Nephrotoma*. Given the size of the genus, a more focused study examining a larger number of species within the genus, examining the WIP of *Nephrotoma* in a phylogenetic context, and use of viewer independent testing would help increase our understanding of WIP at the generic level.

**Figure 9.**
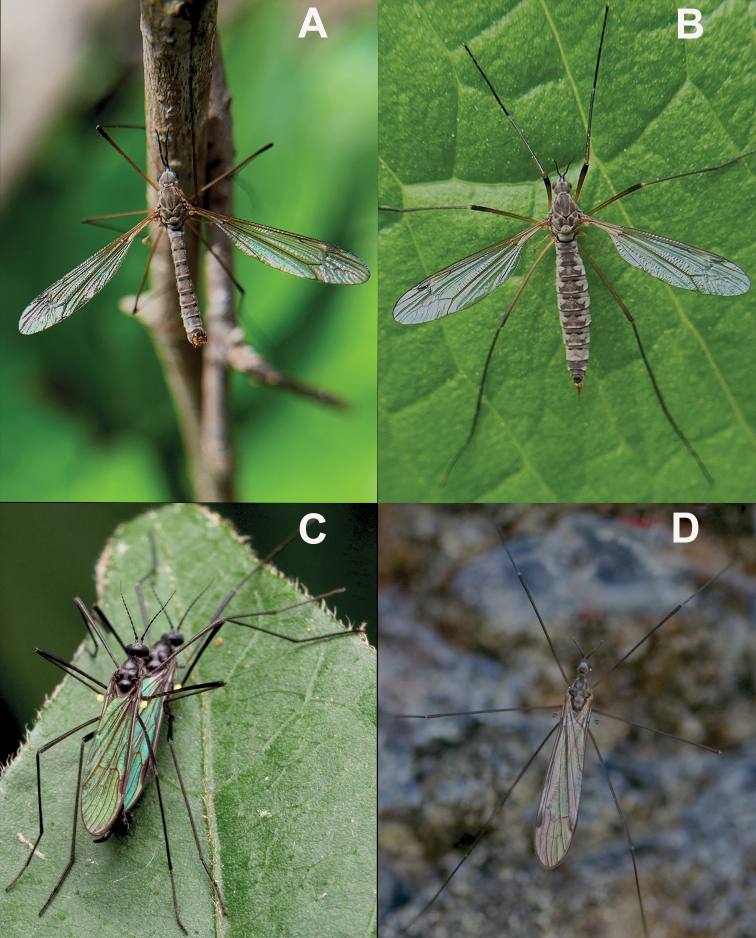
Images showing WIP on several species of crane fly in nature **A** male Tipula (Yamatotipula) aprilina Alexander, 1918 displaying WIP in nature **B** female Tipula (Yamatotipula) aprilina displaying WIP in nature **C** pair of *Gnophomyiatristissima* perched on a leaf in copula. Both flies are displaying their sexually dimorphic WIP. The female (bottom) has a blue WIP while the male (top) displays a green WIP**D** an individual of *Ellipteraclausa* Osten Sacken, 1877 displaying a WIP with wings folded. Sex unknown. Copyright (**A, B**) 2021, photograph JK Gelhaus; (**C**) 2020, photograph Katja Schulz, used with permission by the artist and under a creative commons license (https://creativecommons.org/licenses/by/4.0/) with alterations limited to cropping and resizing of this image; (**D**) 2016, photograph JK Gelhaus. Images are not to scale.

We note that pigmented patterning on the wing appears to reduce the extent of WIP, at least based on our limited survey here. Species with either costal darkening (*Tipulasayi*, *Tricyphonainconstans*) or more extensive marmorated pattern (*Tipulaborealis*, *Dactylolabiscubitalis*), had reduced WIP, at least in the region of the pigment and the pterostigma. Freeman (1968) suggested that marmorated and spotted wing patterns in crane flies are associated with woodland habitats, while clear or striped wings with open habitats; these two different light exposures might impact WIP visibility. Within these broad wing pattern categories, though, there can be a range of wing orientation when at rest which can also impact WIP visibility. For example, *Tipulaborealis* holds the wings outstretched and at a slight angle (Fig. 9A, B), while patterned wing Dactylolabis (Dactylolabis) montana (Osten Sacken, 1860) and Dicranomyia (Dicranomyia) simulans simulans (Walker, 1848) (similar to *Dactylolabiscubitalis*) rest with the wings closed over the abdomen (Fig. 9C, D; Adler and Adler 1991).

Wing interference patterns are stable and they are readily visible in nature (Fig. 9A–D), but their visibility depends on the angle at which light hits the wing as well as the angle it is viewed (Shevtsova 2012). A good example is the male wing of *Dolichopezaobscura* used in this study. When the wing of the male specimen of *D.obscura* is displayed on a white background with light from many different angles it appears clear or slightly stained (Fig. 2) while the same wing, when placed on a dark background with light parallel to the wing displays a bold and intricate WIP (Fig. 5D). Using a pinned museum specimen, we have demonstrated how quickly the WIP transmission can change with a change in background color (see Suppl. material 1: Movie S1). Given this, it is possible that crane flies are using WIP as a type of dynamic flash coloration (Murali 2018) to avoid predation. Additionally, erratic flight patterns have been found to increase the effectiveness of dynamic flash coloration to avoid predation (Murali and Kodandaramaiah 2020) and Pritchard (1983) notes both the wing pattern and the flight pattern of crane flies act to obfuscate the flies from predators. This is merely an observation, and we recommend a true behavioral study to understand what role, if any, WIP plays in predator avoidance strategies in Tipuloidea.

We do remain curious if the WIP in one individual is recognized by other conspecific crane flies. Although WIP were selected for in *Drosophila* (Katayama et al. 2014), in most cases crane flies do not exhibit complicated male-female pre-copulatory behavior (Pritchard 1983), and in many crane flies males seem to find females by touching legs during male search of vegetation (some Limoniidae, TipulidaeStich 1963; Pritchard 1983) or in mating swarms (some Limoniidae, Pediciidae, Alexander 1920; Pritchard 1983). Also, crane flies males search for females or swarm usually during crepuscular periods (Sullivan 1981) or at least early morning and late afternoon (Gelhaus, pers. obs.) when lighting would not be expected to highlight WIPs. Males could potentially recognize a species-specific WIP at close range after initial leg contact, though.

## ﻿Conclusions

The scope of this study was to establish the existence of WIP in the four families of Tipuloidea and confirm that WIP could exhibit sexual dimorphism. We have confirmed stable, structural WIP in male/female pairs of twelve species of crane fly across the four families of Tipuloidea. Of these, eight species displayed sexually dimorphic WIP between male and female specimens. One species showed high intraspecific variation that may be a result of sexual selection, though more research is required. Our work supports the consensus in the literature that WIP are species-specific. This work provides the basis for further research and documentation of WIP in crane flies. We did not compare subspecies in this study and comparisons at the generic level were inconclusive, though we cannot discount a generic level WIP relationship. We believe WIP could be a useful tool to discern cryptic species in crane flies or as a novel character to identify females that cannot be separated based on the current morphology. Additionally, WIP may be used for predator avoidance by crane flies.

## Supplementary Material

XML Treatment for Cylindrotoma distinctissima

XML Treatment for Gnophomyia tristissima

XML Treatment for Dactylolabis cubitalis

XML Treatment for Dicranomyia liberta

XML Treatment for Tricyphona inconstans inconstans

XML Treatment for Dolichopeza obscura

XML Treatment for Brachypremna dispellens

XML Treatment for Holorusia hespera

XML Treatment for Nephrotoma ferruginea

XML Treatment for Nephrotoma macrocera

XML Treatment for Nephrotoma virescens

XML Treatment for Tipula (Beringotipula) borealis

XML Treatment for Tipula (Yamatotipula) sayi

## Supplementary materials

Supplementary material 1 Movie S1 Data type video (mpeg) file Explanation note Movie of pinned male specimen of *Gnophomyiatristissima* with shifting background color to display how WIP can change based on background alone. We created this video in the lab to demonstrate how visual transmission of WIP changes with changes to the background. When the background is white, the wing appears clear but as we move the black background under the wing the WIP is instantly visible. Given this rapid change, we question if this rapid change in WIP could be utilized as an anti-predator defense. Movie created using the rear-facing camera of a LG-G7 cell phone (Model LM-G710TM, Android version 10, software version G710TM30b). https://binary.pensoft.net/file/630017This dataset is made available under the Open Database License (http://opendatacommons.org/licenses/odbl/1.0/). The Open Database License (ODbL) is a license agreement intended to allow users to freely share, modify, and use this Dataset while maintaining this same freedom for others, provided that the original source and author(s) are credited.Robert T. Conrow, Jon K. Gelhaus
